# Annotations

**Published:** 1893-11-11

**Authors:** 


### Annotations.

### Is Pain Necessary?

"I have often wondered," said Dr. Weir Mitchell, addressing the New York Academy of Medicine the other day, "why the great Being, who started us under certain laws upon the long career of reproduction, evolution, and self-modification, should have designed clearly a progress upward, and yet set in our way such limitations. . . . We might to some extent get on without the sense of pain, and that we could do with less of it is shown by the fact that some women remain for years without the pain sense. . . . In one case a hysterical paralytic recovered really useful health except that she cannot be hurt by knife, or fire, or a blow. . . The interior organs still feel pain as usual. This woman used to burn herself from want of care, now she has learned to take heed. . . She doubts whether she would accept anew the natural condition of the pain sense. . . Do we really need it after it has served to give the educational sensory warnings of childhood?" This speculative question is curious rather than practical. That pain has its uses is shown by the fact that Dr. Mitchell's own patient used at first to injure herself by burning and otherwise on occasions when the injuries would have been avoided by the presence of the pain sense. Moreover, she herself is not certain that she would not like to have the pain sense back again. This one fact indeed helps us to an answer to Dr. Weir Mitchell's question. Pain is a ubiquitous and sleepless policeman, acting in our own interests, whose duty it is to compel us to take due care of our bodies and minds. The very intelligent few might, no doubt, after an adequate course of high physiological instruction, get on fairly well without pain. But what of the thoughtless and careless many? Where is there another policeman who does his work so effectually and so economically as pain? Through the long ages of the past, pain has been absolutely indispensable to the progress of the world. If science now proposes to furnish us with a substitute which will play the policeman's part as effectively and more agreeably we have no objection. But until the substitute appears, which shall be as ubiquitous and as inexpensive a preserver of the body and mind as pain, we shall take leave to consider that even this distressful companion of the human species has his decided uses and advantages.

### Diphtheria and Flats.

The alarming idea has been broached that the widespread prevalence of diphtheria in the metropolis may be due to the fœtid airlessness of our modern flats. The public has it on the most unimpeachable authority that about 400 fresh cases a week have been lately reported, and the cases have been of an unusually severe and deadly type. The deaths from diphtheria in the month of October in previous years have generally amounted to about 30. During the October which has just closed the deaths have risen to between 80 and 90, or nearly three times the normal number. When to this is added the fact that there was reported last week an alarming increase in the number of cases of small-pox, we are brought within the range of a series of facts which somebody must look steadily in the face. We know from historical records how deadly have been the epidemics of typhus and other fevers in the old and unventilated flats of the poorer districts of Scotch large towns. But in London, and not in poor districts only, flats are growing in number by hundreds and by thousands, and not in number only but in elevation, in complexity of structure, in the degree of concentration of their residents, and indeed in every circumstance which tends to ruin health, and to raise insanitation to a maximum. Let any person who desires a simple criterion by which to measure the sanitary character of an ordinary London flat, consider the following, for which Sir William H. Broadbent is responsible, and then go and measure the cubic capacity of a block of London flats. Says Sir William Broadbent: "We read with horror such stories as those of the Black Hole in Calcutta, but the effects of over-crowding" (in this country) "are just as destructive, though less speedily so. In the common lodging-houses of the metropolis the police can enforce an allowance of 250 cubic feet of air for each inmate. . . . In workhouses the minimum space of 300 cubic feet is required for each bed. . . . The soldier in barracks has allotted to him a space of 600 cubic feet, 1,200 being allowed in military hospitals." Now let the amateur or professional sanitarian take some of the flats in the metropolis, ascertain their cubic capacity inside, and then calculate the actual number of human beings and animals which occupy the space. He will find in many of our costly London flats that not only is each human being provided with less air space than the 600 feet secured for the soldier in barracks, but actually with less than the 300 feet which is the minimum in workhouse hospitals, or the 250, below which even the casual wards are not permitted to sink. When to this is added the miserable hole-and-corner complexity of structure which characterises so many flats both within and without, the foul and stinking dust-shoots, the cheap and inadequate drainage, and the general stupidity and incapacity of mistresses and servants from a sanitary point of view, it is evident that not only have we here all the requisite elements of an outbreak of diphtheria, but of an overwhelming conflagration of epidemics of every kind. What is to be done? Must we drift until we rush over the brink into the hopeless insanitation of the Middle Ages? Or shall we now make a public and effectual inquiry into the sanitary adequacy of the average London flat as it is actually built and inhabited?

### Secret Remedies and Doctors' Consciences.

Ought the medical profession to be blamed by the public for its abhorence of secret remedies? Mr. Ernest Hart, who is neither narrowminded nor inexperienced, thinks it ought to be praised. Reading men all respect Hippocrates; and they will respect him more if they consider that no less a person than Aristotle was wont to style him "The Great." Now Hippocrates cannot be considered to have been a purist or a medical pedant. What did he think of secret remedies? He thought the dabblers in them so dangerous and so infamous that he administered to every one of his own loyal disciples the famous oath known as the "Hippocratic Oath." That oath Mr. Ernest Hart has made public property in his address to the American medical graduates published in the college number of the *Chicago Clinical Review*. This is the tenor of the "Hippocratic Oath." The disciple swears "to impart the knowledge of his art to others according to the law of medicine; to share with his colleagues all that he knows; to have no medical secret; but to practise his art with purity and holiness." Is it possible that any man can be so blind as not to see that this is an enormous advantage to the public? Can any moralist be so dull witted as not to perceive that it is an enormous gain to good morals? Can anybody fail to see that it raises the capacity of the whole medical profession to the level of the more competent few? How any clever man, any honest man, or any man who takes a broad view of the general interests of mankind can encourage "secret medicine" and their vendors, even in the slightest possible degree, is to us inconceivable. In the court of conscience doctors occupy an absolutely impregnable position in this matter.

